# Krabbe disease: a differential cause of the hyperdense boomerang sign

**DOI:** 10.1055/s-0045-1809997

**Published:** 2025-08-04

**Authors:** Luis Alcides Quevedo Canete, Sérgio Ferreira Alves Júnior, Ângelo Dante de Carvalho Côrrea, Nina Ventura

**Affiliations:** 1 Instituto Estadual do Cérebro Paulo Niemeyer, Rio de Janeiro RJ, Brazil.; 2 Universidade Federal do Rio de Janeiro, Rio de Janeiro, RJ, Brazil.

A 1.5-year-old female child presented with regression of developmental milestones, spastic tetraparesis, and fever. Computed tomography (CT) scans showed hyperdensity, and magnetic resonance imaging (MRI) scans revealed restricted diffusion in the splenium of the corpus callosum, characterizing the boomerang sign ( Figure 1 ). On the follow-up examination, bilateral and symmetrical T2 and fluid-attenuated inversion recovery (FLAIR) hyperintense lesions were observed in the cerebral white matter, predominantly in the parieto-occipital regions, presenting a tiger- or leopard-skin pattern, as well as involvement of the brainstem, corticospinal tracts, and dentate nuclei ( Figure 2 A–C ). Additionally, bilateral thickening and enhancement of the cranial nerves were noted, most prominently in the cisternal portions of the III, V, and VI pairs, and in the intracanalicular portions of the VII and VIII pairs. Diffuse thickening and enhancement of the spinal roots were also observed ( Figure 2 D–G ). Krabbe disease was confirmed through genetic testing, which identified the c.884A>T variant in heterozygosity in the *GALC* gene. In clinical presentations featuring hyperdense lesions on CT and restricted diffusion on MRI in the corpus callosum (splenium), Krabbe disease should be considered. 1 2 3 4

**Figure 1:**
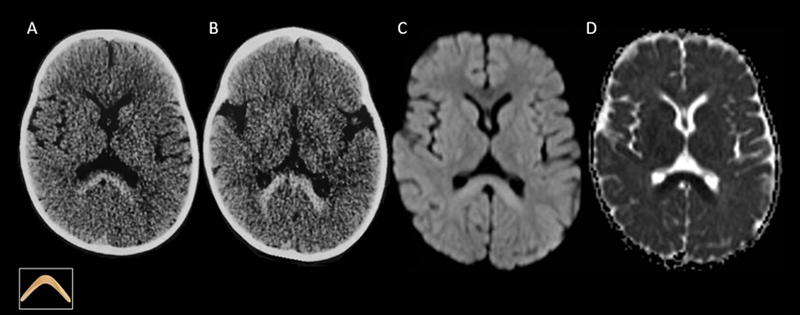
Brain computed tomography (CT) and magnetic resonance imaging (MRI) scans revealing hyperdensity ( **A,B** ) and restricted diffusion ( **C,D** ) in the splenium of the corpus callosum.

**Figure 2:**
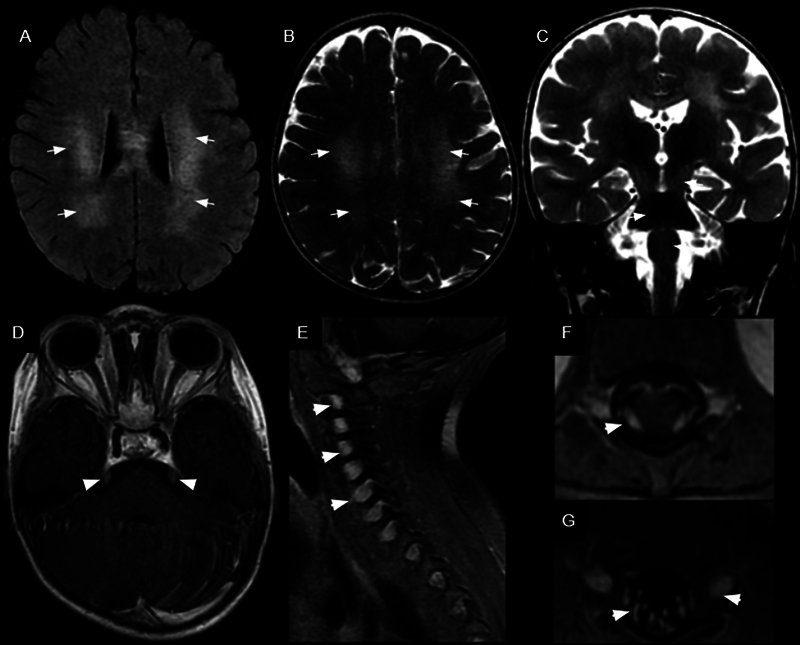
Brain MRI scans revealing bilateral and symmetrical T2 and fluid-attenuated inversion recovery (FLAIR) hyperintense lesions in the white matter of the cerebral hemispheres, predominantly in the parieto-occipital regions, displaying a tiger-like or leopard-skin pattern (white arrows in **A** and **B** ). Involvement of the brainstem and corticospinal tracts (white arrows in **C** ) was observed, along with thickening and enhancement of the trigeminal nerves (white arrowhead in **D** ) and spinal roots (white arrowheads in **E** , **F** , and **G** ).

## References

[1] Muthusamy K Sudhakar S V Thomas M Yoganathan S Christudass C S Chandran M Revisiting magnetic resonance imaging pattern of Krabbe disease - Lessons from an Indian cohort J Clin Imaging Sci 2019 9 25 10.25259/JCIS-18-2019 31448176 PMC6702867

[2] Abdelhalim A N Alberico R A Barczykowski A L Duffner P K Patterns of magnetic resonance imaging abnormalities in symptomatic patients with Krabbe disease correspond to phenotype Pediatr Neurol 2014 50 02 127 134 10.1016/j.pediatrneurol.2013.10.001 24262341

[3] Loonen M C Van Diggelen O P Janse H C Kleijer W J Arts W F Late-onset globoid cell leucodystrophy (Krabbe's disease). Clinical and genetic delineation of two forms and their relation to the early-infantile form Neuropediatrics 1985 16 03 137 142 10.1055/s-2008-1052558 4047347

[4] Bernal O G Lenn N Multiple cranial nerve enhancement in early infantile Krabbe's disease Neurology 2000 54 12 2348 2349 10.1212/wnl.54.12.2348PMID 10881274

